# Pesticide Residues and Unauthorized Dyes as Adulteration Markers in Chilli Pepper and Tomato

**DOI:** 10.1155/2023/5337150

**Published:** 2023-01-13

**Authors:** Edward Ken Essuman, Ernest Teye, Rosemond Godbless Dadzie, Livingstone K. Sam-Amoah

**Affiliations:** ^1^University of Cape Coast, College of Agriculture and Natural Sciences, School of Agriculture, Department of Agricultural Engineering, Cape Coast, Ghana; ^2^University of Health and Allied Sciences, School of Allied Health Sciences, Department of Nutrition and Dietetics, Ho, Ghana; ^3^University of Cape Coast, Africa Centre of Excellence for Food Fraud and Safety, Cape Coast, Ghana

## Abstract

To assess the contamination of processed chilli pepper and tomatoes, a report over the past four decades since the establishment of the Rapid Alert System for Food and Feed (RASFF) was retrieved and analysed. Out of the 887 notification reports assessed for eligibility, 446 were found regarding chilli pepper and tomato contamination. This study identified India as the country of origin with the highest number of reported cases relating to chilli pepper contamination. Italy and Türkiye were the countries with the highest number of reported cases regarding the exportation of adulterated tomatoes to other countries according to the RASFF report. Unauthorized dyes such as Sudan I, III, IV, orange II, rhodamine B, and para red were reported to have been detected in either chilli pepper or tomato in the supply chain. Almost all unauthorized dyes in this study were found to be more than the range (0.5 to 1 mg/kg) of the detection limit of Sudan dye and other related dyes using analytical methods set by the European Union. Unapproved pesticides by the European Union (EU) found in this study were acetamiprid, chlorothalonil, chlorpyrifos, dimethoate, methomyl, monocrotophos, omethoate, oxamyl, and thiophanate methyl. The present study indicates the persistence of chilli pepper and tomato contamination with harmful dyes and pesticide residues despite the ban on the use of certain chemicals in the food chain.

## 1. Introduction

Rapid Alert System for Food and Feed (RASFF) has been recognised as a system for reporting food safety issues within the European Union (EU). It was established in 1979 to ensure the flow of information to enable swift reactions when risks to public health are detected in the food chain. Several research articles have been published using the database of RASFF to report on health risk incidents of the global beef supply chain [1], risk analysis of biogenic amines in food [2], meat products, nut products, fish products [3], food products contaminated with *Listeria* [4], and herbs such as oregano [5]. Condiments used for spices have received relatively little attention and yet play an important role as food ingredients.

Spices, used in whole, broken, or ground forms, are defined as “vegetable products or mixtures thereof, from extraneous matter, used for flavouring, seasoning, and imparting aroma in foods” by the International Standardization Organization [6]. Spices are mostly added in the preparation of food, particularly ready-to-eat food, and are used by consumers as flavouring agents. The safety of food condiments used as spices in the food industry has received little or no attention over the years, although possible contamination of these spices is of key importance to food safety and quality. Most contamination surveys conducted focused on microbes and other secondary metabolites such as mycotoxins. Other contaminants such as chemical contaminants, mainly pesticide residues, are of primary importance in the case of the Capsicum and Solanum families including tomato and chilli pepper [7]. Although spices used in food preparation are in smaller quantities, they add to the contamination levels in the food product when contaminated.

Vegetables such as chilli pepper and tomatoes are high-value food products and play a significant part as constituents in a host of foods. Most foods cannot reach their final preparation stage without the addition of either chilli pepper or tomato. Tomato is a seasonal crop, mostly traded in its fresh state and widely used in soup preparation in the form of a paste, ketchup, concentrate, or juice. It is rich in nutrients such as lycopene, vitamin C, and beta-carotene, which are essential to human health. The annual production of tomatoes on a global scale is approximately 180 million tonnes [8]. Chilli pepper, on the other hand, is a food recipe used all over the world [9], especially in the form of spices due to its sensory attributes such as pungency and colour [10] and is mainly grown in India, China, Mexico, Spain, and Africa. However, according to the Food and Agricultural Organization (FAO) [11], the leading producer of chilli in the world is China (18.9 million tons), followed by Mexico (3.2 million tons), Türkiye (2.6 million tons), Indonesia (2.5 million tons), and Spain (1.4 million tons).

Due to the seasonal nature of chilli and tomato, they are usually processed into powder and other forms to extend their shelf life. In the processing of these food items, the potential presence of accidental substances or deliberate introduction of foreign materials is added. Other chemicals also form part of the product due to poor cultural practices during production. However, the deliberate addition of other substances is for economic gain to increase the bulkiness of the food items; hence, care must be taken when patronizing them [12].

Dyes such as Sudan dye (I, II, III, and IV), para red dye, yellow dye (metanil yellow), and orange II, among others, have been reported to be present in condiments [13]. The use of these dyes is possibly due to their resemblance to the condiment or spices in question since they may be similar in physical or chemical structure. These dyes that are rampantly used by fraudsters are usually not permitted in food processing. One of the most prominent agents regarding the contamination of spices is pesticide residue of agricultural origin [7].

The widespread use of pesticides, herbicides, and insecticides in agricultural production helps to protect crop plants from insects, pests, and pathogens thereby eliminating yield losses. This also improves the quality and quantity of food crops [14]. Vegetables have seen extensive use of pesticides and herbicides in their production due to their increased demand. The report shows that the use of pesticides has warranted almost a third of global crop production [15]. However, the use of pesticides in crop production without good agricultural practices has resulted in the residue of these chemicals remaining on the crops before harvest. The ingesting of such food crops can be dangerous for the individual. A report in 2018 by the European Food Safety Authority (EFSA) shows the presence of pesticide residue in some 91,015 food samples which exceeded the maximum residue level (MRL) and accounted for 4.5% of the overall food sampled [16]. Pesticide residues such as ethion (0.27 mg/kg), acetamiprid (0.12 mg/kg), methomyl (0.06 mg/kg), methamidophos (0.17 mg/kg), acephate, metalaxyl (0.19 mg/kg), profenofos (0.23, 1.2, 0.24, and 0.21 mg/kg), and formetanate (0.18 mg/kg) were reported to have been detected in paprika [7].

Although efforts are being made to dissipate pesticide residues in some food crops [17], its spillover persists. The current study is aimed at reporting on the contamination of chilli pepper and tomatoes over the last 40 years, from 1980 to 2020, using the RASFF portal database.

## 2. Methodology

### 2.1. Data Collection from the RASFF Portal

Information on condiments such as tomato and chilli pepper, which have received very little attention, was retrieved from the RASFF database [18], and this covered the period between 1980 and 2020. The RASFF database is updated daily and is stored on the Communication and Information Resource Centre Administrator (CIRCA) server at the commission. The RASFF serves as an access database that provides information on a specific product such as date of notification, category of contaminants, country of notification and origin, contaminant detection level of the product, and risk decision on the product identified.

**Figure 1:**
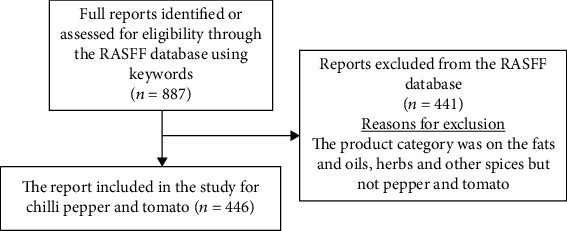
Schematic representation of data identification and exclusion criteria.

Figure 1 shows the number of data retrieved from the RASFF portal and the inclusion and exclusion criteria. Information regarding the contamination of tomato and chilli pepper was based on the following:

1. Product category: herbs and spices
2. Keywords: Sudan dye, tomato, and chilli pepper
3. Notification (country of origin) and hazard types were left clear to include all notifications relating to the product categories

### 2.2. Data Analysis

Data from the dataset that was a product of either tomato or chilli pepper in the form of powder, minced, or crushed were selected and analysed using OriginPro 2021. The data was then presented in tables and figures. The total sum of notifications involving tomato and chilli pepper adulterated with different categories of products was determined. The reported adulterants or contaminants and their respective concentrations in the chilli pepper and tomato samples were summarized and reported in tabular form.

## 3. Results and Discussion

### 3.1. Reported cases per Notifying Country and Country of Origin

In all, 887 notifications were obtained, and the useful ones regarding processed tomato and chilli pepper contamination were selected for analysis. This included 446 notifications. From the time the RASFF started reporting food contamination and adulteration around the world, 244 cases of chilli pepper contamination have been reported (2003 to 2020). Contamination of processed tomatoes stands at 225 cases from the year 1980 to 2020.

Austria recorded 8 cases of chilli pepper contamination and 7 cases of tomato contamination from different countries (Table 1). Three (3) of the chilli pepper cases were from Thailand, and 3 of the tomato cases were from Poland within the period 2003 to 2020.

Belgium recorded 15 cases of contaminated chilli pepper out of which 4 were imported from the Dominican Republic. Tomato contaminated with other foreign materials from other countries to Belgium was 11 and 3 of the cases originated from Belgium itself. No chilli pepper contamination case was recorded in Bulgaria, but 21 cases of contaminated tomatoes were reported, and the majority (9) was from Türkiye.

Very few reported cases of contaminated chilli pepper, and tomatoes were reported in Croatia, Cyprus, Latvia, Luxembourg, Malta, Poland, Romania, Slovakia, Türkiye, Hungary, Ireland, and Lithuania. Nevertheless, Italy (40), the United Kingdom (34), France (33), and Germany (32) recorded the highest notification (in descending order) of contaminated chilli pepper imported into their countries between 2003 and 2020. Germany also recorded 42 cases of contaminated tomatoes, making it the highest notified country, between 1980 and 2020. In this study, Slovakia, Romania, Hungary, Latvia, Malta, and Bulgaria did not record any form of chilli pepper contamination imported into the country. There was equally no recorded case of tomato contamination in Norway, Luxembourg, Türkiye, and Lithuania.

Regarding the number of times notified countries have reported cases of contaminated chilli pepper, Italy recorded 5 times from India, 10 times from Egypt, and 7 times within Italy. The United Kingdom recorded 17 times from India and 6 times from Italy. Germany recorded 5 times from India, Finland recorded 5 times from Thailand, and France recorded 12 times from the Dominican Republic, all within the period between 2003 and 2020, as shown in Table 1. Among the notified countries that reported cases of contaminated chilli pepper, Germany was the country that recorded the highest case of importation of contaminated chilli pepper from 18 countries.

Tomato contamination as reported by the RASFF and notified by Bulgaria was from Türkiye (13 times) and Jordan (8 times) in the period 1980 to 2020. The other notifying countries are as follows: Germany recorded 9 times from Türkiye and 22 times from Italy; Greece recorded 9 times from Türkiye; Denmark recorded 6 times from Italy; the Netherlands recorded 5 times from its own country; Italy recorded 7 times from its own country and 5 times from Türkiye; and Belgium recorded 5 times from Italy. The notifying countries with the highest number of contaminated tomato cases were Italy (9) and Germany (9) from 1980 to 2020.

In all, the country of origin with high chilli pepper contamination was India (50 times) followed by Dominican Republic (29 times) and Thailand (27 times). Italy (56 times) and Türkiye (53 times) were the reported countries with high exportation of contaminated tomatoes to other countries according to the RASFF report as shown in Table 1.

**Figure 2:**
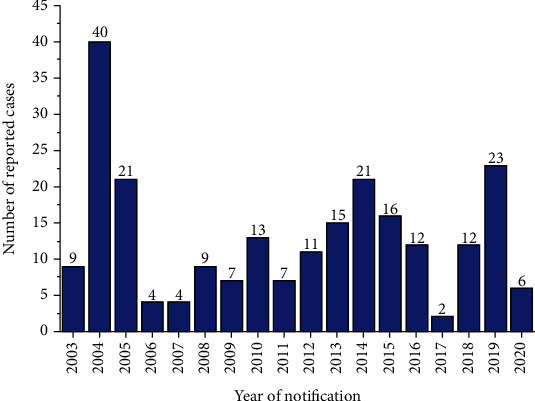
Number of notification reports per year for chilli pepper adulteration/fraud within the period 2003 to 2020.

**Figure 3:**
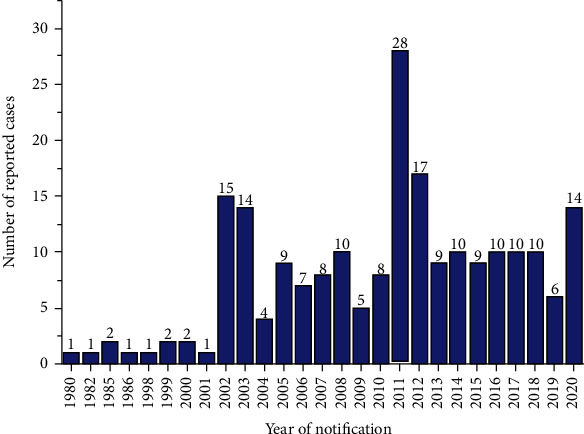
Number of notification reports per year for tomato adulteration/fraud within the period 1980 to 2020.

Contamination of chilli pepper, as reported by the RASFF portal, started in 2003. In 2004, 40 cases of contaminated chilli pepper (Figure 2) were recorded from around the world as reported in this study. The high number of recorded cases in 2004 was due to food authorities in other countries embarking on control programmes which resulted in increased RASFF notifications. The actual RASFF notification peaked at 270 cases in 2004 which included chilli pepper, other spices like curry powder, and food items such as sauces, red palm oil, and pasta [19]. In this study, the reported cases dropped to 2 in 2017 and rose to 23 in 2019. Contamination of processed tomatoes was first reported in 1980 by the RASFF portal with cases ranging from one to two between 1980 and 2001 (Figure 3). A rise in the contamination of tomato cases was reported in 2011, and this dropped to 6 in 2019. The variation in processed chilli pepper and tomato contamination discovered in this study could be attributed to rising food demand caused by a rapidly growing population, as well as the unfavourable spillover effect of food adulteration from other countries [20].

### 3.2. Classification of Hazards for Chilli Pepper and Tomato as Reported by RASFF

The current study exposed the contaminants of processed tomato and chilli as reported by RASFF from 1980 to 2020. The broad nature of the contaminants or adulterants reported to be present in tomato and chilli pepper was categorized into pesticide residues, composition, mycotoxins, food additives, and so on as shown in Table 2. A total of 104 and 94 pesticide residues were reported to have been found in processed chilli pepper and tomato, respectively, over the period. Indeed, pesticide residues feature at the top of this study concerning hazard types found in tomato and chilli pepper and are usually among the top issues for products from nonmember countries. Compositions in the form of an unauthorized dye such as Sudan I, III, and IV, rhodamine B, and orange II were reported to have been found in processed chilli pepper and tomato (Table 3). Mycotoxins and microbial contamination were also reported to have been found in chilli pepper and tomatoes. Food spoilage resulting from microbial contamination may be due to the infusion of different microbes through several sources. The contamination reported in this study might have come about during the processing of tomato and chilli pepper like harvesting, handling, preparation, distribution, and storage. Although the hazard posed by contamination in spices applies to dynamic contamination capable of multiplication, microbial contamination, or related substances, particularly of pathogenic microorganisms, static contamination by chemical substances such as pesticide residues cannot be ignored [7].

Foreign materials such as glass fragments, metal fragments, and infestation of mould were found only in tomatoes. Metals such as lead, cadmium, chromium, and tin were found in tomatoes together with other migrations of di-(2-ethylhexyl) phthalate (DEHP) (Table 2). The presence of different types of metals found in tomato and chilli pepper could be classified as an incidental form of adulteration. According to Bansal et al. [21], metals such as mercury, cadmium, and lead are considered very toxic and their intake could be chronic as these metals are associated with organ damage. Packaging defects of processed tomato and chilli pepper such as bulging, absence of labelling, or incorrect labelling were suspected to be a result of contamination. Other forms of hazards identified include environmental pollutants, allergens, organoleptic aspects (deterioration), food additives, and flavourings.

**Figure 4:**
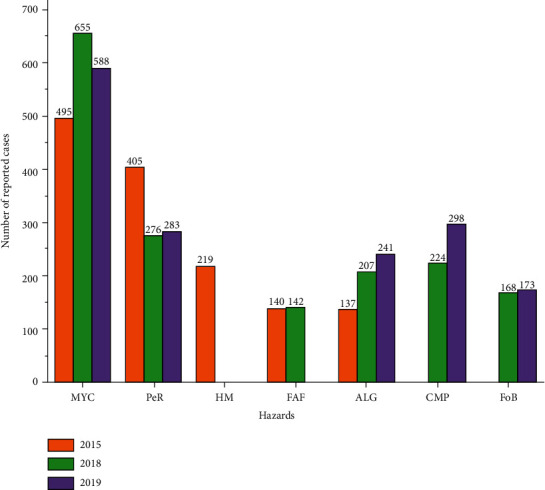
Hazard notification trends as reported by the RASFF annual report. Source: RASFF annual report [28, 38, 39]. MYC = Mycotoxins; PeR = Pesticide residues; HM = Heavy metals; FAF = Food additives and flavourings; ALG = Allergens; CMP = Composition; FoB = Foreign bodies.

Following the trend of hazard notification as reported in the RASFF annual report, most of the hazards reported in food and its products by notifying countries keep soaring.  Figure 4 shows the trend of hazards as reported in 2015, 2018, and 2019 by the RASFF annual report.

### 3.3. Colourants Used to Adulterate Chilli Pepper and Tomato

Unauthorized colours such as orange II, Sudan dye, rhodamine B, fast garnet, oil orange, and para red were reported to have been found in either processed chilli pepper or tomato. Although these colours were banned to be used in food products, high quantities were reported to be present in either chilli pepper or tomatoes. Orange II in chilli pepper and tomato was in the range of 0.50 to 30.00 mg/kg as shown in Table 3. The amount of rhodamine B reported being present in chilli pepper and tomato ranges from 0.44 to 140.00 mg/kg. Para red was the least colourant reported to have been found in chilli pepper and tomato with an average value of 0.7 and 0.4 mg/kg, respectively. The increased presence of para red in chilli pepper than tomato was also reported by Mustafa et al. [13] in estimating para red dye in chilli powder and tomato sauce. The findings in this study agree with Rao et al. [22] who also reported the presence of soluble dye and rhodamine in chilli powder.

**Figure 5:**
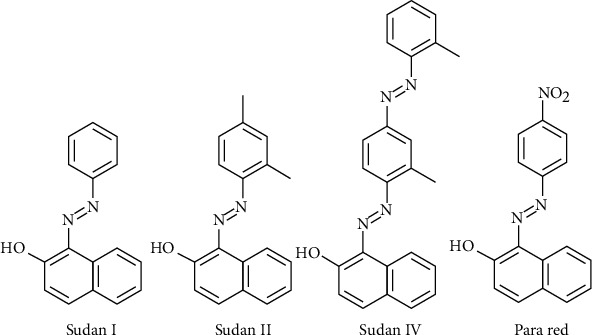
Structure formulae of some common names of azo dyes under study [19].

Sudan III, Sudan IV, fast garnet, and oil orange Ss were not found in tomatoes (Table 3). The reason could be attributed to the fact that the Sudan dye is red in colour and tomato in either processed form does not look reddish; hence, the addition of Sudan dye will make it look suspicious to consumers. However, these dyes belong to the azo family of synthetic dyes widely used as colouring agents in the cloth industry. They have been categorized under class 3 carcinogens by the International Agency for Research on Cancer [23]. According to the European Union and Food Standards Agency, Sudan dye and other related dyes belonging to the azo family are banned to be used as additives to improve colour in foodstuffs for human consumption or commercial benefit [24] because they could pose health issues to the consumer due to their toxic and carcinogenic nature. Sudan I in hot chilli pepper was first reported by the French food control in 2003, and an alert was posted by the EU in the RASFF [25]. This led the EU to ensure that chilli pepper and chilli products imported into member states should be accompanied by an analytical report demonstrating that the consignment does not contain Sudan dye [19]. A detection limit of 0.5 to 1 mg/kg of Sudan dye and other related dyes (see structures in Figure 5) for the analytical method was set by the European Commission [26], but what was observed in this study for chilli pepper and tomato was far more.

Many food manufacturers use these dyes or colourants to cover the ageing effect, mask spoilage, and disguise poor or bad foodstuffs or processed products [13]. The quantity and the type of dye being used are therefore not important to the manufacturer besides achieving their aim.

### 3.4. Pesticide Residues Present in Chilli Pepper and Tomato

**Figure 6:**
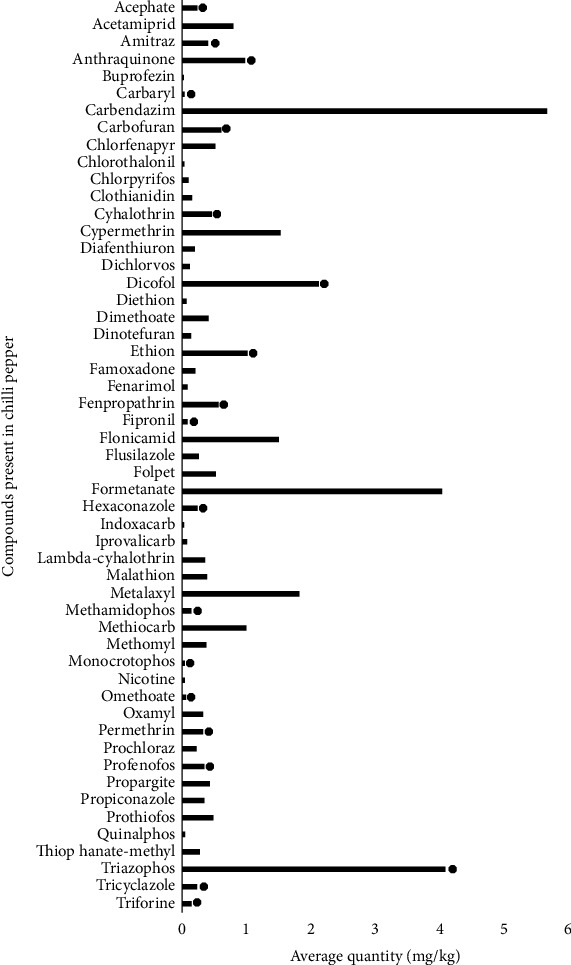
Pesticide residue levels reported by RASFF of the European Union in chilli pepper.

**Figure 7:**
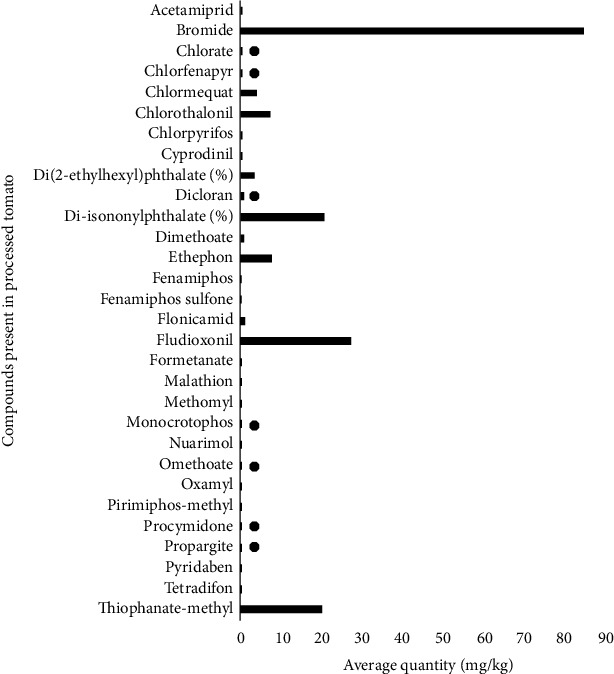
Pesticide residue levels reported by RASFF of the European Union in tomato.

This study shows evidence of the use of unapproved pesticides. The substances marked with ● are not authorized in the EU at the time of this study as shown in Figures 6 and 7. This includes acetamiprid, chlorothalonil, chlorpyrifos, dimethoate, methomyl, monocrotophos, omethoate, oxamyl, and thiophanate methyl which were found in both chilli pepper and tomato. Carbendazim, formetanate, and triazophos (5.69, 4.05, and 4.11 mg/kg, respectively) were some of the chemical compounds with high residual levels reported to have been found in chilli pepper (Figure 6). The highest pesticide residues reported to be present in processed tomatoes were thiophanate methyl (20.26 mg/kg), fludioxonil (27.36 mg/kg), Di-isononyl phthalate (20.70%), and bromide (84.9 mg/kg) as shown in Figure 7. On tomato surface, other substances like thiabendazole, carbendazim, and chlorpyrifos have been found [27]. The most frequently reported pesticide residues in chilli pepper and tomato and the number of notifications can vary from year to year [28]. Besides pepper and tomatoes which are used as spices, other types of products that have been found to contain pesticide residues as reported by the RASFF annual report [28] include cereals and bakery products, cocoa and cocoa preparations, coffee and tea, fruits, and vegetables.

**Figure 8:**
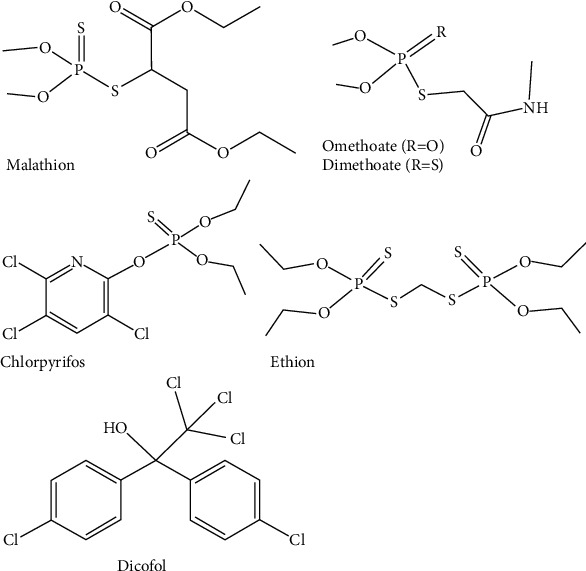
Pesticide active ingredients of the organophosphate and organochlorine groups reported by RASFF of the European Union in processed chilli pepper and tomato.

Recent studies show that some of the pesticides reported in this study are highly dangerous to human health and the environment especially pesticides in the chemical groups of organophosphates (malathion, chlorpyrifos, dimethoate, dichlorvos, cypermethrin, and ethion, see Figure 8) and organochlorine (dicofol) [29, 30]. The number of pesticide residues reported in this study is due to misuse, overuse, improper application of pesticides to the crops, illegal use of pesticides not registered and authorized for crops, and inadequate harvest or storage conditions (waiting period of the last pesticide application) [29]. Again, most countries do not have guidelines for highly dangerous pesticide use [31]. Therefore, the use of such crop commodities as raw materials for food production becomes a reasonable source for the occurrence of pesticide residues in processed foods as reported in this study.

The official limits for pesticide residues including condiments used for spices can be found in the Codex Alimentarius [32].  Table 4 provides information on some tolerable levels of pesticides that are currently used in the food production chain, and some of these compounds were reported in this study as shown in Figures 6 and 7. However, the established maximum residue limits (MRLs) are based on national conditions and practices and are possible for different countries to set different MRLs for the same pesticide or food commodity. Hence, it is likely that a pesticide legal in one country is not authorized for use in another country where it is not considered necessary for pest control. This, therefore, results in unfair barriers to trade.

According to Röösli et al. [29], the use of unauthorized pesticides not only disturbs the soil conditions but also destroys the biocontrol agents in the soil which need to be cared for and developed through the reduction of chemical usage in agriculture. Humans get exposed to pesticides found in food through ingestion and dermal contact [29]. Exposure to pesticides has been linked to many health problems such as diminished intelligence, immune suppression, reproductive abnormalities, hormone disruption, cancer, and neurological and behavioural disorders, especially among children [29, 33, 34]. The Food and Agricultural Organization (FAO) [35] reported that billions of people in the world depend on agriculture for their livelihoods, hence the increase in exposure to pesticide usage. The presence of pesticide residues in crops has affected the importation and exportation of crops from one country to another country [29].

As revealed in this current study, the unauthorized pesticides and colours are mostly cheap; hence, farmers indulge in the act to get them and indiscriminately apply them to their crops. Again, due to the high demand for tomatoes, most producers do not wait for the recommended safe period of the pesticides they use before the crops are harvested for market and export. Some eco-friendly measures that can be adopted to reduce pesticide residue on crops include spraying the crops with a mixture of water extract from the bark of *Tamarindus indica* fruit and leaves of the neem tree [29, 36, 37]. This helps to control fungal infections without any adverse effects on the crops.

To minimize these forms of contamination, there is a need to adopt good agricultural practices (GAP) before and after harvesting agricultural produce. The presence of pesticide residues found in these vegetables is mostly due to over and indiscriminate doses of synthetic fertilizers and pesticide application. Although some foods may contain natural toxins, the presence of unauthorized pesticide residue can pose more danger to the consumers' health. This needs to be replaced by the need-based application of safe and recommended pesticides as this will help reduce chemical use in general. Before exporting food items to other nations, food safety authorities in the country of origin should conduct routine surveillance, monitoring, inspection, and random sampling of food products, including processed tomatoes and chilli pepper. Food Safety Officers of the State can create quick tests for the identification of adulterants, particularly banned colours, so that citizens can identify adulteration in their own homes and raise consumer awareness of food safety.

## 4. Conclusion

The findings of this study indicate that the exportation of contaminated processed chilli pepper and tomato is common. Unauthorized dyes such as orange II, Sudan dye, rhodamine B, fast garnet, oil orange, and para red were reported to have been found in either processed chilli pepper or tomato. Carbendazim (5.69 mg/kg) and bromide (84.9 mg/kg) were some of the chemical compounds with high residual levels reported to have been found in processed chilli pepper and tomato, respectively. The levels of contaminants reported in this study are alarming despite the ban on certain dyes and pesticides used in the food chain. There should be more rigorous and routine testing of products originating from countries to help curb the menace by providing risk managers with a sound evidence base for designing future monitoring programmes.

## Figures and Tables

**Table 1:** RASFF data of adulteration reported in the chilli pepper and tomato supply chain by notifying country and country of origin.

Notifying country	Year of case	Chilli pepper		Year of case	Tomato	
		Country of origin	Number of reports		Country of origin	Number of reports
Austria	2019	Italy	2	2020	Türkiye	1
	2015	Thailand	1	2016	Poland	1
	2014	Thailand	2	2015	Poland	1
	2011	Tunisia	1	2014	Poland	1
	2003	Türkiye	1	2010	Türkiye	1
	2003	Italy	1	2010	Germany	1
				2003	Germany	1
Belgium	2020	Cameroon	2	2018	Italy	1
	2020	Nigeria	1	2016	Türkiye	1
	2015	Gambia	1	2014	France	1
	2015	Dominican Republic	1	2007	Belgium	3
	2015	Thailand	1	2005	Italy	1
	2014	Thailand	1	2004	Italy	1
	2014	Dominican Republic	1	2004	Netherlands	1
	2013	Dominican Republic	2	2003	Italy	1
	2010	Thailand	1	2000	Italy	1
	2009	Uganda	1			
	2008	Thailand	1			
	2008	India	1			
	2003	Italy	1			
Bulgaria				2013	Türkiye	2
				2012	Türkiye	5
				2011	Türkiye	4
				2011	Jordan	8
				2011	Türkiye	2
Croatia	2016	India	1	2018	Italy	1
				2017	Albania	1
				2017	Türkiye	1
Cyprus	2017	Sri Lanka	1	2017	Sri Lanka	1
	2012	India	1	2011	Greece	1
	2010	Egypt	1			
Czech Republic	2018	India	1	2017	Morocco	1
	2016	Vietnam	1	2017	Italy	1
	2015	Laos	1	2017	Netherlands	1
				2016	Poland	1
				2014	Morocco	1
				2014	France	1
				2008	Netherlands	2
Denmark	2020	Türkiye	1	2020	Austria	1
	2018	Thailand	1	2020	Italy	1
				2019	Sweden	1
				2012	Italy	1
				2011	Italy	2
				2010	Türkiye	1
				2010	Switzerland	1
				2009	Italy	1
				2006	Spain	1
				1980	Brazil	1
				1980	Italy	1
				1980	Portugal	1
Finland	2018	Türkiye	1	2020	Poland	1
	2014	Mexico	1	2013	Finland	1
	2013	Thailand	3	2008	Türkiye	1
	2013	Spain	1	2005	Germany	1
	2013	India	1	2005	Türkiye	1
	2012	Thailand	1	2003	Italy	1
	2009	Thailand	1			
France	2020	Vietnam	1	2020	Morocco	1
	2019	Dominican Republic	1	2020	Spain	1
	2019	Vietnam	1	2018	Germany	1
	2018	Vietnam	1	2018	Spain	1
	2018	Dominican Republic	1	2016	Italy	1
	2016	India	1	2014	Morocco	2
	2015	Dominican Republic	4	2013	Morocco	1
	2014	Dominican Republic	2	2012	Morocco	1
	2014	Lebanon	1	2011	France	2
	2014	Netherlands	2	2010	Spain	1
	2013	Thailand	1			
	2013	India	1			
	2012	India	1			
	2012	Dominican Republic	3			
	2010	Dominican Republic	1			
	2010	India	1			
	2005	Spain	1			
	2004	South Africa	1			
	2004	France	5			
	2004	India	1			
	2004	Lebanon	1			
	2003	India	1			
Germany	2019	Thailand	1	2020	Italy	1
	2018	Thailand	1	2018	Italy	2
	2018	Vietnam	1	2016	Italy	1
	2015	India	1	2014	France	1
	2015	Dominican Republic	1	2014	Tunisia	1
	2012	Dominican Republic	1	2014	Italy	1
	2012	United Kingdom	1	2013	Spain	1
	2012	China	1	2013	Italy	1
	2012	South Korea	1	2012	France	1
	2012	Türkiye	1	2011	Italy	1
	2010	Pakistan	1	2011	Germany	1
	2009	United Arab Emirates	1	2010	Italy	2
	2008	Chile	1	2010	Morocco	1
	2006	Belgium	1	2010	France	1
	2005	China	1	2008	Greece	1
	2005	India	4	2008	Türkiye	1
	2005	Pakistan	2	2007	Türkiye	3
	2005	Ukraine	1	2006	Türkiye	1
	2005	Austria	1	2005	Türkiye	3
	2005	Belgium	1	2003	Türkiye	1
	2005	Unknown	3	2003	Italy	1
	2004	Türkiye	1	2002	Netherlands	1
	2004	Lithuania	1	2002	Spain	2
	2004	Egypt	1	2002	Italy	10
	2003	Thailand	1	1985	Italy	2
	2003	Netherlands	1			
Greece	2007	Unknown	1	2020	Italy	1
	2004	India	4	2020	Türkiye	1
	2004	Unknown	1	2019	Italy	1
				2018	Hungary	1
				2017	Egypt	1
				2012	Türkiye	2
				2011	Türkiye	1
				2011	Georgia	1
				2009	Georgia	2
				2009	Greece	1
				2008	Syria	1
				2007	Türkiye	1
				2006	Türkiye	1
				2003	Türkiye	1
				2001	Türkiye	1
				2000	Türkiye	1
Hungary				2020	Türkiye	1
				2020	Hungary	1
				2011	Spain	1
				2010	Türkiye	1
Ireland	2008	India	1	2017	Ireland	1
				2005	Italy	1
Italy	2019	India	1	2020	Italy	2
	2018	Dominican Republic	1	2019	Bangladesh	1
	2018	Bangladesh	1	2019	Türkiye	3
	2017	Egypt	1	2017	Egypt	2
	2016	Egypt	1	2015	Tunisia	2
	2016	Vietnam	1	2006	Romania	1
	2016	India	1	2006	Tunisia	1
	2015	Sri Lanka	1	2006	Cuba	1
	2014	Dominican Republic	1	2005	Italy	2
	2014	Pakistan	1	2005	Türkiye	1
	2014	India	1	2004	Tunisia	1
	2013	India	1	2003	Albania	1
	2013	Bangladesh	1	2003	Türkiye	1
	2013	Cambodia	1	2002	Italy	1
	2011	Pakistan	1	1999	Syria	1
	2010	Thailand	1	1986	Italy	1
	2009	Peru	1	1982	Italy	1
	2008	Tunisia	1			
	2007	Cameroon	1			
	2007	Egypt	1			
	2005	Italy	3			
	2005	Egypt	1			
	2005	India	1			
	2004	Spain	1			
	2004	Egypt	6			
	2004	United Arab Emirates	2			
	2004	Pakistan	1			
	2004	Italy	4			
	2004	Tunisia	1			
Latvia				2018	Türkiye	1
				2009	Türkiye	1
				2006	China	1
Lithuania	2004	China	2			
	2004	Poland	3			
	2004	India	2			
Luxembourg	2015	Thailand	1			
Malta				2018	Italy	1
Netherlands	2019	United Kingdom	2	2020	Netherlands	2
	2019	India	1	2016	Spain	2
	2015	India	1	2016	Netherlands	1
	2014	France	1	2015	Germany	2
	2008	Thailand	2	2015	Morocco	1
	2006	Thailand	1	2014	Spain	1
	2004	Türkiye	3	2012	Netherlands	1
	2004	Germany	1	2008	Netherlands	1
Norway	2016	Thailand	1			
	2015	Vietnam	1			
	2014	Cambodia	1			
	2014	Vietnam	1			
	2013	Vietnam	1			
	2006	Thailand	1			
	2006	Türkiye	1			
	2005	Syria	1			
Poland	2008	China	1	2015	Ukraine	1
	2008	Netherlands	1	2011	China	1
				2011	Egypt	1
				2011	Morocco	1
Romania				2012	Bulgaria	2
				2012	Netherlands	2
				2011	Türkiye	1
				2011	Bulgaria	1
Slovakia				2016	Hungary	1
Slovenia	2020	India	1	2012	Slovenia	2
	2019	Egypt	1	2012	Macedonia	1
	2012	Egypt	1	2008	Slovenia	1
				2004	Bulgaria	1
Spain	2019	Uganda	1	2013	Mexico	1
	2016	Morocco	1	2003	Türkiye	2
	2014	China	1	2003	India	1
	2011	Dominican Republic	2	2002	Türkiye	1
	2010	Bolivia	1			
	2010	Dominican Republic	3			
	2009	Bolivia	1			
	2005	Spain	2			
Sweden	2019	Laos	1	2015	Italy	1
	2016	Thailand	1	2013	Spain	1
	2016	India	1	2013	Netherlands	1
	2010	India	1	2011	Morocco	1
	2009	Thailand	1			
	2003	Lebanon	1			
Switzerland	2014	Vietnam	1	2018	Türkiye	1
	2013	Thailand	1	2014	Italy	1
	2013	Egypt	1			
	2013	Türkiye	1			
Türkiye	2019	Belgium	2			
United Kingdom	2019	India	3	2016	Ghana	1
	2019	Pakistan	2	2012	Italy	1
	2019	Dominican Republic	1	2010	Ireland	1
		Uganda	2	2008	Italy	3
	2018	India	2	2007	Morocco	1
	2018	Dominican Republic	1	2003	Portugal	1
	2016	India	2	2003	Türkiye	1
	2015	India	1	1999	Italy	1
	2014	Dominican Republic	1	1998	Italy	1
	2014	India	3			
	2013	Egypt	1			
	2011	India	3			
	2010	Dominican Republic	1			
	2010	Ireland	1			
	2009	Türkiye	1			
	2007	Malaysia	1			
	2005	Pakistan	1			
	2005	India	1			
	2004	United Arab Emirates	2			
	2004	India	1			
	2004	Poland	1			
	2004	Pakistan	1			
	2003	India	1			

**Table 2:** Categories of hazards for chilli pepper and tomato identified as reported by the RASFF portal (1980 to 2020).

Hazard types	Chilli pepper		Tomato
Composition	92		6
Pesticide residues	104		94
Mycotoxins	26		2
Foreign bodies	—		19
Food additives and flavourings	1		28
Metals	—		6
Migration	—		15
Microbial contamination (moulds/others)	4		22
Allergens	—		3
Adulteration/fraud	3		1
Organoleptic aspects (deterioration)	1		8
Environmental pollutants	1		4
Packaging defective	—		6

**Table 3:** Unauthorized dyes present in chilli pepper and tomato as reported by RASFF.

Composition type	Chilli pepper	Tomato
	Range (mg/kg)	Range (mg/kg)
Orange II (uc)	>0.50-30	30
Sudan 1 (uc)	0.022-5468	0.01-9.35
Sudan III (uc)	0.655-14.7	—
Sudan IV (uc)	0.01-2105	—
Rhodamine B (uc)	0.4413-140	140
Fast garnet (uc)	0.77	—
Oil orange Ss (uc)	0.15	—
Para red (uc)	0.661	0.4

uc = unauthorized colour.

**Table 4:** Pesticide residues and their maximum residue limits (MRLs).

Chemical compounds	MRLs (mg/kg)
Acetamiprid	1
Bromide	20.0
Buprofezin	0.5
Chlorfenapyr	1
Chlorothalonil	0.5
Chlormequat	0.05
Chlorothalonil	0.5
Chlorpyrifos	0.05
Clothianidin	1
Cypermethrin	0.03
Cyprodinil	5.0
Diafenthiuron	0.02
Dichlorvos	0.1
Dimethoate	1
Ethephon	2.0
Famoxadone	0.02
Fenamiphos	0.02
Fenarimol	1.0
Malathion	8.0
Metalaxyl	1.0
Methiocarb	0.05
Methomyl	3.0
Pirimiphos-methyl	0.1
Prochloraz	2.0
Propargite	3.0
Propiconazole	0.05
Pyridaben	1.0
Quinalphos	0.02
Tetradifon	1.0
Thiophanate-methyl	2.0

Source: http://apeda.gov.in/apedawebsite/Announcements/.

## Data Availability

Some of the data can be obtained from RASFF Portal (https://webgate.ec.europa.eu/rasff-window/portal/?event=SearchByKeyword~~~~~~~~~^~^~^~^~~~~~~~~~~~amp;NewSearch=1~~~~~~~~~^~^~^~^~~~~~~~~~~~amp;Keywords=sudan%204) (accessed 02/03/2021) and also http://webgate.ec.europa.eu/rasff-window/screen/search (use the following keywords: Sudan IV, tomato, and pepper). Consult the corresponding authors for the extracted data from the RASFF database in Excel format.
